
JOURNAL INFORMATION
==============================
NLM Title Abbreviation: Med Chir Rev
NLM Journal ID: 0034617

The Medico-Chirurgical Review

Publisher: Biomedical Journal Digitization Project

ARTICLE INFORMATION
==============================
PMCID: PMC4966184
PMID: 29917606
Article version: 1
Subjects: Reviews

Dr. Abercrombie on Apoplexy

Print publication date: 1828 Jul 1
Volume: 9
Issue: 17
First page: 73
Last page: 86
1828 May 24
License: This work is free of known copyright restrictions. For more information, please see PMC Back Issue Digitization.
License URL: https://creativecommons.org/publicdomain/mark/1.0/

==============================

### VIII.

*Pathological and Practical Researches on Diseases of the Brain, &c.* By John Abercrombie, M.D.

[Apoplexy.]

We do not much wonder that the routine practitioner should have as great a horror of pathological investigations, as had the Turkish Cadi of hearing two sides of a question. These Researches are admirably calculated to disturb settled opinions—to weaken fixed principles—and, in short, to engender scepticism, and undermine the foundations of medical faith. The routine practitioner has one name for each disease—one or two leading or pathognomonic symptoms—one, or, at the most, a couple of causes—but a whole magazine of remedies. In such a pleasant state of things, pathology is a most unwelcome intruder. It is like the doubt of "Providence's sway" which rose suddenly in the mind of Parnell's hermit, and by which "all the tenor of his hopes was lost." It is like the fragment of rock which rolls from the aerial summit of a cliff, and plunges into the tranquil lake below, whose glassy bosom, the moment before, reflected clearly and distinctly every feature of the surrounding and superincumbent scenery, celestial and terrestrial, while—

————— glittering fragments of a broken sun,
Banks, trees, and skies, in thick disorder run.

Analogous to this are the modern investigations into the connexion between outward symptoms and physical phenomena, on the one hand, and internal disorders of function, or change of structure, on the other. These investigations leave no hope that medical practitioners shall, in future, repose on beds of roses. Their confidence in a few diagnostic symptoms must be broken—their reliance on a routine administration of drugs, for the cure of diseases of which they are often ignorant, must be withdrawn, or greatly diminished—and, finally, medical men of every denomination must now study or starve! This is evidently the opinion of the distinguished author, whose work is now before us. He shews us candidly the difficulties that surround us, but urges these very difficulties as the best reasons for renewed exertions.

"In the investigation of the cases which are more properly referable to the head of apoplexy, we find the same difficulties which have met us in the inflammatory affections. A person previously in perfect health falls down suddenly, deprived of sense and motion, and dies, after lying for some time in a state of coma. We find on examination a large coagulum of blood compressing the surface of the brain, or filling its ventricles, and the phenomena of the disease appear to be distinctly accounted for. Another person is cut off with the same symptoms, and we expect to find the same appearances, but nothing is met with except serous effusion, in no great quantity, in the ventricles, or only on the surface of the brain. A third is seized in the same manner, and dies, after lying for a considerable time in a state of coma, from which nothing can rouse him for an instant; and on the most careful examination, we cannot detect in his brain the smallest deviation from the healthy structure."

Dr. A. observes, that we have seen the same want of uniformity in regard to various other morbid conditions of the brain. "We are not, however, to sit down contented with this profession of our ignorance, but ought, on the contrary, to consider this conclusion as a position of much importance, from which we are to commence a new course of investigation." It is to be recollected, however, that in pathology, as in all other branches of medical science, we must act according to the *general results* of observation and experience—not according to the exceptions, however numerous they may be, which we meet in our investigations. Thus, in the passage which we have quoted from Dr. Abercrombie, he tells us that, in one man who dies with the usual symptoms of apoplexy, we shall find a sanguineous effusion in the head—but that, in another, we may not be able to detect any trace of physical lesion in the brain. Dr. A. however, has omitted to tell us that, in perhaps 30 cases of apoplexy, with the usual symptoms, we shall not fail once to meet with the proper physical lesions after death. If, therefore, in the 31st case, we should be disappointed in our endeavours to detect the cause of the disease, we are not, on that account, to distrust the symptoms that are presented in the next patient under our care. But to proceed with our proper analytical labours.

Dr. A. tells us, that the most simple illustration of apoplexy is derived from those cases which result from mechanical causes, as tight neck-cloths, strangulation, &c.—also those cases where people fall down suddenly, in a state of perfect apoplexy, and yet speedily recover, without any remaining paralysis. In these cases, Dr. A. remarks, "the apoplectic attack must be supposed to depend upon a cause, which acts simply upon the circulating system of the brain, producing there a derangement which takes place speedily, and is often almost as speedily removed." Now here we must differ in the very outset, and on a very important point of pathology, from our talented author. Why, in the name of physiology, should not the brain, the sensorium commune, be affected through the medium of its nerves, as well as other organs? We see the heart's function violently disturbed—nay, arrested, by a moral emotion:—We also see the function of the same organ disturbed and interrupted by irritation of a nerve in the stomach or bowels. Now all this must be through the medium of the nervous system. Yet Dr. A. does not seem disposed to admit that the functions of the brain (the centre of the nervous system) can be disturbed or interrupted, as in these cases of transient apoplexy, except through the agency of the circulation simply. In the absence of direct proof, then, we have every reason to believe that, in the cases in question (excluding strangulation, and other mechanical causes) the affection of the nervous system is the primary, as well as the principal pathological condition—and that, whatever derangement of the circulation may obtain in these sudden and transitory attacks of apoplexy, it is subsequent, secondary, or, in other words, the effect of the nervous derangement. Dr. A. indeed, acknowledges that it is very difficult to say in what this simple derangement of the circulation consists—since we have "no data upon which it can be determined by legitimate deduction." The wise course, therefore, is to correctly ascertain those symptoms which *most generally* obtain during life, and those organic changes which most commonly correspond with them in the dead body. The exceptions to general rules should be known, and borne in mind, but they should rarely influence our conduct in the general routine of our practice. But we must proceed to our analytical labours.

Our author remarks that the apoplectic attack is generally preceded by symptoms indicative of deranged circulation in the brain, such as headach, giddiness, sense of fulness, pulsation, noise in the ears, &c. The intellectual functions sometimes evince slight aberrations, and the senses are disturbed. Transient paralyses of certain muscles are occasionally observed, as in speech, walking, &c. The attack itself occurs under three forms, which are thus delineated by our talented author.

"I. In the first form of the attack, the patient falls down suddenly, deprived of sense and motion, and lies like a person in a deep sleep; his face generally flushed, his breathing stertorous, his pulse full, and not frequent, sometimes below the natural standard. In some cases convulsion occurs, in others rigid contraction of the muscles of the extremities; and sometimes contraction of the muscles of the one side, with relaxation of the other. In this state of profound stupor, the patient may die after various intervals, from a few minutes to several days; or he may recover perfectly, without any bad consequence of the attack remaining; or he may recover from the coma, with paralysis of one side. This paralysis may disappear in a few days, or it may subside gradually, or it may be permanent. Other functions, as the speech, may be affected in the same manner, being speedily or gradually recovered, or permanently lost; and recovery from the apoplectic attack is sometimes accompanied by loss of sight.

"II. The second form of the disease begins with a sudden attack of pain in the head; the patient becomes pale, sick, and faint; generally vomits, and frequently, though not always, falls down in a state resembling syncope; the face pale, the body cold, and the pulse very feeble. This is sometimes accompanied by slight convulsion. In other cases, he does not fall down, the sudden attack of pain being only accompanied by slight and transient loss of recollection. In both cases he generally recovers in a few minutes from the first effects of the attack, is quite sensible and able to walk, but continues to complain of headach; after a certain interval, which may vary from a few minutes to several hours, he becomes oppressed, forgetful, and incoherent, and then sinks into coma, from which he never recovers. In some cases paralysis of one side occurs, but in others, and I think the greater proportion of this class, no paralysis is observed.

"III. In the third form, the patient is suddenly deprived of the power of one side of the body, and of speech, without stupor; or if the first attack be accompanied by a degree of stupor, this soon disappears; he seems sensible of his situation, and endeavours to express his feelings by signs. In the farther progress of this form of the disease great variety occurs. In some cases, it passes gradually into apoplexy, perhaps after a few hours; in others, under the proper treatment, the patient speedily and entirely recovers. In many cases the recovery is gradual, and it is only at the end of several weeks or months that the complaint is removed. In another variety, the patient recovers so far as to be able to speak indistinctly, and to walk, dragging his leg by a painful effort, and after this makes no farther improvement. He may continue in this state for years, and be cut off by a fresh attack, or may die of some other disease without any recurrence of the symptoms in his head. In a fifth variety, the patient neither recovers, nor becomes apoplectic; he is confined to bed speechless and paralytic, but in possession of his other faculties, and dies gradually exhausted, without apoplexy, several weeks or months after the attack.

"These three forms of disease frequently pass into one another; but they are very often met with, as they are here described, forming affections which differ remarkably from each other; and they appear very naturally to arrange themselves into the three classes which have here been referred to;—first, those which are immediately and primarily apoplectic; secondly, those which begin with a sudden attack of headach, and pass gradually into apoplexy; thirdly, those which are distinguished by palsy, and loss of speech, without coma."

A section is next dedicated to a consideration of those cases that are primarily apoplectic—in which cases we find the patient sometimes speedily recover—sometimes die, presenting extravasation of blood, or effusion of serum—"in many cases no morbid appearance whatever." The latter, which Dr. A. designates as simple apoplexy, seems to him to establish the important fact, that there is "a modification of apoplexy depending upon a cause of a temporary nature, without any real injury done to the substance of the brain—that the condition upon which this attack depends, may be removed almost as speedily as it was induced—and that it may be fatal without leaving any morbid appearance in the brain." This is an undoubted fact, and Dr. Abercrombie need not have related so many cases from his own and other men's practice, to establish it. As our author assimilates this class of cases with that in which serous effusion is found, he goes on to state a series of cases of the latter description, which we shall pass over, of course, as not necessary for the pathological investigation that is to follow. We shall introduce an extract touching what has been termed serous apoplexy, and the distinction, real or imaginary, between it and sanguineous apoplexy.

"I. The distinction which has been proposed betwixt sanguineous and serous apoplexy is not supported by observation. The former is said to be distinguished by flushing of the countenance and strong pulse, and by occurring in persons in the vigour of life; the latter by paleness of the countenance and weakness of the pulse, and by affecting the aged and infirm; and much importance has been attached to this distinction, upon the ground, that the practice, which is proper and necessary in the one case, would be improper or injurious in the other. I submit that this distinction is not founded upon observation; for, in point of fact, it will be found, that many of the cases which terminate by serous effusion, exhibit, in their early stages, all the symptoms which have been assigned to the sanguineous apoplexy; while many of the cases, which are accompanied by paleness of the countenance and feebleness of the pulse, will be found to be purely sanguineous; and one modification of the disease in particular will be described, in which these symptoms are very strikingly exhibited, while the disease is found to be sanguineous apoplexy in its most hopeless form. Portal has described a series of cases which afford the same result; of three, which presented all the symptoms of serous apoplexy, one was saved by repeated bleeding, and in the other two which were fatal, there was found extensive extravasation of blood. Case XC. lately described, forms a remarkable addition to these observations. If any case could be confidently considered as serous apoplexy, this was such. Dropsical effusion had existed in the body for months; and in defiance of every remedy, it had been progressively gaining ground. There were symptoms indicating its existence both in the thorax and in the abdomen; the patient then became comatose, with pale countenance, and died; but though dropsy was found in the other cavities, none could be detected in the brain.

"II. In other parts of the body, serous effusion is very seldom a primary disease; it arises as a result, either of inflammatory action, or of impeded circulation, and takes place slowly, not accumulating at once in such quantity as to induce urgent symptoms. It is, therefore, in the highest degree improbable, that it should occur in the brain as a primary disease, and accumulate with such rapidity as to produce the symptoms of an apoplectic attack.

"III. The quantity of fluid effused bears no proportion to the degree of the apoplectic symptoms. We find it in small quantity though the apoplectic symptoms had been strongly marked and long continued; we find it in large quantity when the symptoms have been slight; and, finally, we find most extensive effusion in the brain where there have been no apoplectic symptoms at all. The direct inference from these facts is, that in the cases of apoplexy with effusion, the presence of the fluid cannot be considered as the cause of the apoplectic symptoms.

"The facts which have been related in this section appear to warrant the following conclusions.

"1. There is a modification of apoplexy which is fatal, without leaving any morbid appearance that can be discovered in the brain.

"2. There is another modification, in which we find serous effusion often in small quantity.

"3. The cases which are referable to these two classes are not distinguished from each other, by any such diversity of symptoms as can be supposed to indicate any essential difference in their nature.

"4. Without any apoplectic symptoms, we find serous effusion in the brain in an equal or in a greater quantity than in the cases of the second modification.

"5. It is therefore, probable, that in these cases the effusion was not the cause of the apoplectic symptoms.

"6. It is probable, that the cases of the first modification depend upon a cause which is entirely referable to a derangement of the circulation in the brain distinct from inflammation.

"7. It is probable, that the cases of the second modification are, at their commencement, of the same nature with those of the first; and that the serous effusion is to be considered as the result of that peculiar derangement of the circulation, which constitutes the state of simple apoplexy. In other words, it is probable, that the affection which has been called serous apoplexy is to be considered as simple apoplexy terminating by effusion."

Previously to entering on any speculations respecting the nature of apoplexy, we must follow our able author through some sections on practical subjects.

*Cases not primarily apoplectic.*—The prominent symptoms in these cases are, a sudden attack of violent headach—the patient often starting up and screaming from its severity. Sometimes he falls down pale, faint, and exhausted, occasionally with a slight convulsion, from which he recovers, in a short time, so far as to be able to walk home, the symptoms going on under various modifications—especially the headach, and sickness at stomach.

"The patient continues for some time, perhaps an hour or two, cold and feeble, with cadaverous paleness of the countenance; his pulse weak and generally frequent. He is quite sensible, but oppressed. By degrees he recovers heat and the natural appearance of the countenance, and the pulse improves in strength. The face then becomes flushed; he is more oppressed; he answers questions slowly and heavily; and at last sinks into coma, from which he never recovers. The period occupied by these changes varies exceedingly in different cases. In one case to be described, there intervened, from the first attack to the commencement of coma, 5 hours; in a second, 12 hours; in a third, 3 days; and in a fourth, not more than 15 or 20 minutes. Death followed the appearance of coma in the first of these cases in 7 hours, in the second in 32 hours, and in the third in two days."

In Dr. A.'s experience, these cases are generally fatal—forming a modification remarkably different from the simple apoplectic state—" and, on inspection, we find none of those varieties and ambiguities which occur in the apoplectic cases, but uniform and extensive extravasation of blood." He thinks we are justified in concluding, that they depend upon the immediate rupture of a considerable vessel, without any previous derangement of the circulation, " the rupture arising from disease of the artery at the part which gives way."

"At the moment when the rupture occurs, there seems to be a temporary derangement of the functions of the brain, but this is soon recovered from. The circulation then goes on without interruption, until such a quantity of blood has been extravasated as is sufficient to produce coma. In their whole progress, these cases are strictly analogous to those of extravasation on the surface of the brain, from external injuries. The patient recovers from the immediate effects of the injury, walks home, and after some time, perhaps an hour or two, becomes oppressed, and at last comatose. The extravasated blood being in this case removed by the operation of trephine, the coma disappears. The varieties of the symptoms which occur in this form of the disease, are such as we might expect upon this view of the nature of the affection. In some cases it is probable that the extravasation goes on progressively, until such a quantity has been accumulated as is sufficient to produce the fatal coma. In others, there is reason to believe that soon after the rupture has taken place, the hæmorrhage is stopped by the formation of a coagulum, and after a considerable interval bursts out afresh and is fatal."

A variety of cases are here introduced by our author, in illustration of the foregoing observations. These we must pass over.

The source of the hæmorrhage, in cases of this class, is extremely various. Perhaps the most common is a rupture of a moderately sized vessel in the substance of the brain, whence the blood makes its way into the ventricles, or to the surface—or in both directions at the same time. There are cases, however, where the blood appears to spring from the minute capillary vessels, by what we might almost term exhalation. This is the meningeal apoplexy of Serres, and other Continental pathologists. In some instances, the hæmorrhage has been distinctly traced to ulceration and rupture of one of the principal arterial trunks—in others, to the vessels of the choroid plexus—to rupture of the sinuses—of small aneurisms, &c.

In respect to the diseased conditions of vessel in the substance of the brain, to which Dr. A. attributes the hæmorrhage, ossification, or earthy brittleness is one. The calibre of the artery is often lessened at the hardened part—sometimes entirely obliterated.

"In other cases again, numerous branches of the principal arteries of the brain will be found to present a succession of small opake osseous rings, separated from one another by small portions of the artery in a healthy state. This is a very common appearance in the brains of elderly people, and the rings are generally from half a line to a line in breadth, and are separated from each other by healthy portions of about the same extent. In some cases again, the inner coat of the artery is much thickened, of a soft pulpy consistence, and very easily separated; so that, when a portion of the artery is compressed between the fingers, a considerable quantity of this pulpy matter is forced out."

Dr. A. thinks that this diseased condition is probably the cause of a variety of complaints in the head, which ultimately prove fatal by the rupture of vessels.

*The Paralytic Cases.* Under this head our author described cases which resembled each other in symptoms only, but were widely different in their pathology. We are not yet possessed of sufficient facts to enable us to discriminate these cases from one another, in a satisfactory manner. The leading phenomenon, in this class, is the paralytic attack without coma.

"The attack appears under various forms; the most common of which is hemiplegia with loss of speech; but in some cases the speech is not affected; while in others, the loss of speech is at first the only symptom. In some cases again, one limb only is affected, which is most commonly the arm, though sometimes the leg. Numerous other modifications occur, as, palsy of one eyelid; or of the orbicularis of the eye; distortion of the eyes; double vision; twisting of the mouth, &c. Loss of the power of swallowing also occurs occasionally, though more rarely in the cases which do not pass into apoplexy. The patient frequently appears quite sensible of his situation, and makes anxious attempts to express himself; generally understanding in a great measure what is said to him, and answering by signs. Sometimes, however, when he retains a partial power of speech, his answers are incoherent and unintelligible.

"In the farther progress of cases of this class, we observe remarkable varieties which may be chiefly referred to the following heads.

"I. Such an attack may be merely the prelude to the apoplectic, and may pass into it after a short interval. These cases belong chiefly to the second class.

"II. The attack may, under the proper treatment, pass off speedily and entirely, leaving, after a very short time, no trace of its existence.

"III. The recovery may be very gradual, the use of the affected limbs being restored after several weeks or months.

"IV. The palsy may be permanent; that is, the patient, after a certain time, may recover so far as to be able to walk about, dragging his leg with a painful effort, and to speak very imperfectly; and after this, makes no farther improvement to the end of his life, which may be protracted for many years.

"V. In a fifth variety the patient makes no recovery; he is confined to bed, speechless and paralytic, but possessed of his other faculties in a considerable degree, and dies gradually exhausted, after several weeks or months; in some cases without coma, in others with coma for a few days before death.

"In endeavouring to investigate the morbid conditions of the brain which are connected with these varieties, we find considerable diversities, which, in a pathological point of view, may be referred to the following heads.

"I. Many of the cases appear to have a close analogy to simple apoplexy; and when they are fatal, present either no satisfactory appearance, or only serous effusion, often in small quantity.

"II. Extravasation of blood of small extent, contained in defined cysts in the substance of the brain or under the membranes.

"III. Ramollissement of the cerebral substance.

"IV. Inflammation and its consequences."

When a formidable paralytic attack passes off speedily and entirely, without leaving any trace of disease, we may conclude that no very serious injury has been sustained by the brain, and that mere turgescence of vessels, or sympathy with some other organ, had been the pathological condition. Many cases again are fatal, and present, on dissection, only serous effusion, often in small quantity.

"I have formerly given my reasons for believing, that serous effusion in apoplectic cases is not a primary disease, but a termination of simple apoplexy; and I have described cases in which it existed to a considerable extent without paralysis. In the cases, again, in which it has been accompanied by paralysis, the quantity of fluid has borne no proportion to the symptoms, and has been equally distributed over the brain; except in the case now quoted from Morgagni, in which too, it is worthy of remark, it appeared to be most abundant on the same side with the disease. From these considerations, I think we may conclude, that, in the cases now referred to, the effusion was not the cause of the paralysis, but the effect or the termination of a certain state of the circulation in a part of the brain, with which the paralysis had been connected from the first invasion of the disease. The whole phenomena of palsy, do indeed bear evidence, that certain cases of it depend upon a cause, which is of a temporary nature, and capable of being very speedily and entirely removed. We see hemiplegia take place in the highest degree, and yet very rapidly disappear; but the most singular circumstance, connected with certain cases of palsy, is, that we occasionally see it continue without any improvement for many weeks or months; and then, from some change which entirely eludes our observation, take a turn for the better, and very suddenly disappear."

In respect to paralytic cases dependent on extravasations of blood in the brain, we find that, when the cerebral substance surrounding the clot continues in a healthy state, the coagulum, even of large size, will be gradually and completely absorbed in time. The process of absorption commences soon, but goes on very slowly. In fourteen or fifteen days from the extravasation, the character of the coagulum is considerably changed. The thinner parts have disappeared, and the remainder has become firmer in texture. At last it assumes the appearance of a fibrous texture—the dark red colour disappears, and a small mass of fibrine only remains. Even this, in process of time, almost entirely vanishes.

"While these changes are going on in the coagulum, the cavity in which it is contained becomes lined with a distinct firm membrane of a yellowish colour; and when the coagulum has entirely disappeared, we find the cyst remaining, and forming a distinct and well defined cavity, which is generally entirely empty. Bands of the same yellow matter which forms it are frequently seen crossing it in various directions, and producing a connexion betwixt its opposite sides at these points. The French writers believe that the cyst is at last entirely obliterated; but I have never seen any thing to satisfy me that it is so. I have examined such cysts at various periods of their progress, but have seen nothing like an approach to obliteration, except the bands which I have now mentioned, connecting their opposite sides. The cyst appears to be distinctly organized, and numerous bloodvessels have sometimes been observed ramifying upon it."

As the absorption goes on, the symptoms, in some cases, gradually subside and entirely disappear—but, in others, the improvement is but partial, and the coagulum is sometimes removed, the paralysis remaining for life. In some, the symptoms appear to go off speedily, though there is every reason to believe that the coagulum remained unabsorbed.

*Softening of the Brain.* Dr. Abercrombie recurs to this subject, and endeavours to corroborate the conjecture previously offered, that the disorganization in question is analogous to gangrene in other parts of the body—and that, like gangrene, it may arise from two different causes.

"These are inflammatory action, and failure of circulation depending upon disease of the arterial system. Ramollissement arising from the former cause I conceive to be an affection which holds a prominent place in the pathology of acute affections of the brain, occurring in early life, and being accompanied by symptoms of an acute character. I have given my reasons for considering this as an affection in many respects distinct from the ramollissement, investigated with such care by the French pathologists, and particularly M. Rostan. This appears to be a disease of the aged,—the cases described by M. Rostan, having chiefly occurred in persons from 70, to 80, and upwards. It is accompanied by symptoms of a paralytic and comatose character, and is frequently complicated with extravasation of blood. I have already alluded to the frequency and the extent of the disease of the arterial system of the brain in advanced life; and there appears to be considerable probability in the conjecture, that this may be the source of the ramollissement in the cases of this class. The disease of the arteries consists of ossification, with thickening and contraction, frequently to a great extent, and sometimes with separation of the inner coat. It corresponds precisely with the state of the arteries, which we know to produce gangrene in other parts of the body, particularly in the toes and feet of old people; and, in another place, I have described a remarkable case, in which separation of the inner coat of the iliac artery produced gangrene of the whole extremity, which was fatal in four days.

"The symptoms accompanying the ramollissement, in this form, do not appear to differ remarkably from those which occur in the other cases of palsy. The attack is frequently preceded by some of the usual symptoms indicating a tendency to disease of the brain; such as, headache, giddiness, partial loss of recollection, and numbness or prickling of some of the limbs. After some time, there occurs suddenly or more gradually a loss of power of one or more limbs, with embarrassment of speech, but without loss of intelligence. The patient is then confined to bed, helpless and oppressed, with more or less of palsy, but with a certain degree of intelligence; and dies after various intervals, either with gradual sinking, or with coma for a few days before death, or sometimes with symptoms of low fever. The symptoms seem frequently to advance more slowly than in the cases from extravasation, one organ, as the tongue, being first affected, and then one or more limbs after some interval. Pain in the affected limbs is also a frequent occurrence; and rigid contraction of them has been much insisted on, as a symptom characteristic of the ramollissement. It is, however, by no means a uniform symptom, for it is frequently wanting altogether; when it is present, in the early period of the disease, it frequently disappears in the course of it; and, in some of Rostan's cases, it left the limbs of the paralytic side and affected those of the other. It is also frequently observed in cases entirely of another kind—as in certain states of fever which terminate favourably. Convulsive affections of the limbs are also occasionally met with.

"The patient is frequently, in the early stages, quite intelligent, and observant of every thing, although unable to speak, or able to speak but imperfectly. As the disease advances, he frequently becomes more oppressed and somnolent; but he is intelligent when roused, not entirely comatose till the last period, and sometimes never entirely so. In other cases, there is at first a considerable degree of coma, which after the first day goes off, the patient becoming quite intelligent, and giving appearances of recovery, which are found to be fallacious. The sensibility of the affected limbs is in some cases impaired, in others little affected; and, in some cases, they are found acutely painful. The ramollissement is of various extent. It seems to be most commonly seated in the more external parts of the brain, near the surface, but sometimes is found in more deep-seated parts, particularly in the corpus striatum. A small coagulum of blood is sometimes found involved in the softened part; and sometimes the softening is found to have taken place around the cyst of an old extravasation."

It is curious that this softening, in an uncombined state, is much more frequently met with in the French than in the English hospitals.

### GENERAL PATHOLOGY OF PARALYSIS.

Dr. A. acknowledges that there are great difficulties attendant on the investigation of this subject, since we find paralysis connected with so many different affections of the brain—and all these affections so frequently existing without paralysis. We must be contented with drawing such conclusions as the general tenor of our observations may authorise. Thus we find paralysis connected with the following morbid appearances:—

"I. Simple and recent inflammation of the cerebral substance.
"II. This inflammation passing into ramollissement.
"III. The encysted abscess of the brain.
"IV. Induration of a portion of the cerebral substance.
"V. Extravasated blood in the ventricles; on the surface of the brain; and in cavities or cysts in the substance of the brain.
"VI. The empty cysts from which extravasation has been absorbed.
"VII. Serous effusion on the surface of the brain.
"VIII. Extensive disease of the arteries of the brain."

Some other points are here touched upon by our author, not arising immediately out of the preceding classification. The first of these is loss of a considerable portion of the cerebral substance, a very remarkable instance of which is recorded by Dr. O'Halloran, where, from the destruction, by accident, of the frontal bone, a large suppuration took place—immense masses of brain came away—and "a frightful cavern" was left. On the 8th day, there was paralysis of the left hand and arm—on the 10th, of the left thigh and leg. The man lived till the 17th day, retaining his faculties till the last, and without any disturbance of the vascular system. The second instance is from COLD. A man is mentioned by Dr. Clerk, who became paralytic in both legs, and partially in the arms, after being benumbed with cold on the top of a coach. He recovered by aid of mercury and the warm bath. Dr. A. has seen a case of paraplegia referred to this cause, and which has now lasted nearly three years, with little amendment.

"Dr. Powel has described three cases of paralysis of one side of the face, producing great twisting of the mouth, and in one of them, inability to shut the eyelids. The affection came on immediately after exposure to cold, by a cold wind blowing upon one side of the face; it was not accompanied by any other symptom; two of them were well in eight or ten days; but the third, a child, was not free from the complaint for three months. They seemed to derive benefit from sudorifics and the application of steam."

We wonder Dr. A. did not see that the above were cases of local affection of the portio dura, now so well understood by all attentive practitioners, and immediately afterwards adverted to by the Doctor himself. We need not dilate on this topic.

Dr. A. alludes to a modification of paralysis, apparently connected with the state of the circulation in the affected part. A lady (mentioned by Dr. Storer) was recovering from pneumonia, when, one morning, she was suddenly seized with acute pain in the left shoulder, extending to the arm, at the same time the whole left side became paralytic, the parts were cold, and the arteries of the limbs were without pulsation. On the right side the pulse was natural. For some days she seemed to be improving, but she died before any pulsation returned in the parts affected. She was not examined. We cannot advert to any more of the anomalous examples brought forward by our author, but proceed, at once, to the important subject of Therapeutics.

### Outline of the Treatment of Apoplexy.

" From the facts which have been related, we have seen reason to believe, that there is a modification of apoplexy which is fatal without leaving any morbid appearance, and which probably depends upon a deranged condition of the circulation in the brain; we have also seen grounds for believing, that the cases which terminate by effusion are probably at their commencement in this state of simple apoplexy. We have seen farther, that we have no certain mark by which we can ascertain the presence of effusion; and finally we have found, that even extensive extravasation of blood in the brain may be entirely recovered from by the absorption of the coagulum. These considerations give the strongest encouragement to treat the disease in the most active and persevering manner. They teach us, also, not to be influenced in our practice, by the hypothetical distinction of apoplexy into sanguineous and serous; and finally, not to be hasty in concluding in any particular case, that the disease has passed into a state in which it is no longer the object of active treatment.

" In the treatment of apoplexy, our remedies are few and simple. Those in which our chief reliance is to be placed are, large and repeated blood-letting, active purgatives, and cold applications to the head, aided by an elevated position of the body, cool air, and the absence of all stimuli. Antimonials may occasionally be useful as an auxiliary, from their known effect in restraining vascular action, provided in the early stages they do not occasion vomiting.

" Our first great object is to take off the impulse of blood from the arteries of the head, by bleeding carried to such an extent as shall powerfully and decidedly affect the system, and by repeating it at short intervals as soon as these effects begin to subside. The first bleeding should probably be from the arm, but, after this, there seems to be an evident advantage in abstracting blood locally either from the temporal artery or by cupping. Much importance has been attached by some to bleeding from the jugular vein, as most likely to give immediate relief to the head; but we must remember, that the only jugular vein that can be opened is the external jugular, which has very little communication with the brain, and consequently that bleeding from it is probably much inferior to bleeding from the temporal artery. As soon as possible after the bleeding, means are to be taken for inducing strong purging. The most efficient remedy by far for this purpose is the croton oil, and, if the patient cannot swallow, it may be very conveniently introduced into the stomach, suspended in thick gruel or mucilage, through an elastic gum tube; the operation should be expedited by strong purgative injections. This is always to be considered as a most important and leading point in the treatment of apoplexy; and though, in arresting the progress of the disease, our first reliance is upon large and repeated bleeding, the first decided improvement of the patient is generally under the influence of powerful purging. The effectual application of cold to the head is the third remedy on which we rely, and it is equally applicable to the different states of the disease, whether arising from simple apoplexy or from extravasation. It may be applied either by means of iced water, or pounded ice in a bladder; or by a full stream of cold water directed against the crown of the head, and received in a basin held under the chin, while the patient is supported in a sitting posture. I have formerly given an example of a patient restored in a few minutes or rather seconds by this remedy from a state of perfect apoplexy.

"The active use of these remedies is sometimes followed by a very speedy removal of the apoplectic state. In other cases, though little immediate effect may be produced, yet, by a persevering repetition of them, the coma begins to subside after some time, perhaps a good many hours, or even several days. In other cases again, they may be used in the most active manner without relieving the patient, and after all we may find upon dissection, that the disease was still in the state of simple apoplexy. This important fact cannot be too often repeated or too carefully kept in mind; it should lead us to prosecute the treatment of every case of apoplexy with the utmost vigour and perseverance. In one of the last cases that occurred to me, the bleeding was repeated to the extent of upwards of one hundred ounces, assisted by purging from the croton oil, repeated to the extent of above twenty drops, and the case terminated favourably, after the symptoms had continued in a very doubtful state for three or four days.

"In the extent of our evacuations, indeed, a due regard is certainly to be had to the age and constitution of the patient, and the strength of the pulse; but I think we have sufficient ground for saying, that there are no symptoms which characterize a distinct class of apoplectic affections, requiring any important distinction in the treatment; or in other words, a class, which in their nature do not admit of blood-letting. On this important point, we may refer with some confidence to the facts which have been related. Weakness of the pulse, and paleness of the countenance, we have seen to be frequent symptoms of the worst form of sanguineous apoplexy; and on the other hand we have seen cases terminate by serous effusion, which were accompanied by strong pulse and flushing of the countenance. Finally, we have seen one remarkable case in which there existed every circumstance that could lead us to consider the disease as serous apoplexy, but which was fatal without any effusion; and another in which there was most extensive effusion without any apoplectic symptom. It is likewise to be kept in mind, that in apoplectic affections the strength of the pulse is a very uncertain guide, for nothing is more common than to find it upon the first attack of apoplexy, weak, languid, and compressible, and becoming strong and full after the brain has become in some degree relieved by large blood-letting."

Dr. A. details some cases occurring in old and infirm people, which were treated on the foregoing principles, though their ages and constitutions might have seemed to forbid any measures of the kind. We shall glance at one or two of these en passant.

A woman, aged 70, of spare habit, and thin withered aspect, fell down in the street speechless, and paralytic of the left side. Dr. A. saw her in four or five hours afterwards. She was oppressed, but not entirely comatose—she was entirely speechless and paralytic—pulse 96, and tolerably strong. She was bled to 15 ounces—purgatives were exhibited—and cold was applied to the head. The following day, she was something better—but, getting worse in the evening, she was again freely bled, and purgation was continued. At the end of a week she was able to walk with assistance—and in a few days more she was quite well.

The following case we shall give in our author's own words.

"A gentleman, aged 70, of a spare and feeble habit, and very infirm from frequent attacks of asthma, without any warning fell from his chair on the floor in a state of *perfect apoplexy*, accompanied by violent *convulsion*. When I saw him, an hour after the attack, he was still in a state of perfect coma; the convulsion had recurred at short intervals, and had affected chiefly his arms and his face. His face was pale; his pulse was of good strength and a little frequent. He was largely bled from the arm; and an active purgative was given, assisted by a purgative enema, and cold was applied to his head. The convulsions continued for some time to recur with great violence; they then became less severe, and at length ceased about three hours after the attack, leaving him in a state of coma. But the purgative having soon after operated freely, he recovered his recollection. Next day, he complained of headach, and took more purgative medicine; and after a few days more he was in his usual health."

We have always thought that it was an error to define a disease to be "perfect apoplexy," where there is "violent convulsion." The definition—or, at least, the essential pathognomonic features of apoplexy, are, loss of *sense and motion*. How, then, can we reconcile perfect apoplexy with convulsions? To us it appears that the combination in question should be termed "APOPLEXIA EPILEPTICA," and not perfect apoplexy.

We have now considerably exceeded the limits which we originally chalked out for this department of Dr. Abercrombie's work. The practical nature of the publication, and the talents of the author were seductions, which, like the apples of Atalanta, constantly drew us aside from the *end* which we had in view. These *digressions*, we hope, will be pardoned both by author and reader. We shall again have occasion to pay our respects to Dr. Abercrombie, when on the subject of spinal diseases.

In an appendix to the part on apoplexy, Dr. Abercrombie has added some interesting observations, modestly entitled "CONJECTURES," in regard to the circulation in the brain. The brain, we know, is peculiarly situated, in consequence of the bony and unyielding box in which it is contained. When, therefore, a person dies of apoplexy, and we can discover no extravasation or congestion, we are naturally led to ask ourselves what was the pathological condition that caused death? We know that when animals are bled to death, their brains exhibit either a natural appearance, or the vessels unnaturally turgid with blood. From the situation of the brain, it is probable that no state of things can materially augment or diminish the actual amount of fluids within the cranium.

"The cranium is a complete sphere of bone, which is exactly filled by its contents, the brain, and by which the brain is closely shut up from atmospheric pressure, and from all influence from without except what is communicated through the blood vessels which enter it. In an organ so situated, it is probable, that the quantity of blood circulating in its vessels cannot be materially increased, except something give way to make room for the additional quantity, because the cavity is already completely full; and it is probable, that the quantity cannot be materially diminished, except something entered to supply the space which would become vacant."

All this we are ready to grant; but the relative proportions of blood circulating in the arterial and venous systems of the brain may, from circumstances, be greatly changed. Thus the arterial system may become surcharged—with a corresponding diminution of quantum in the venous system. It is clear that this change of balance in the circulation of the brain must cause unequal pressure, or even disruption of situation in certain parts of the brain, the effects of which may be serious, or even fatal. Thus the portions of brain in the tracts of the large arterial trunks must be injuriously pressed upon, when these vessels are inordinately dilated, and the same with the parts contiguous to veins, when it happens to be their turn for turgescence. But, independent of any material change of balance in the arterial and venous circulation of the brain, it must be remembered that, even if no more blood could be pushed into the vessels at one time than at another, still the *attempt* to push in more—namely the *vis à tergo* acting on the arteries, or interruption to the return of blood from the veins, must cause *pressure* on the brain generally—and this pressure is, in our minds, quite sufficient to account for the sudden deaths which we sometimes see in apoplexy, without any *post-mortem* appearances to account for the fatal termination. This *pressure* Dr. A. appears determined not to see—or not to acknowledge. But we shall not urge the business further in this place. The following practical observations are deserving of record, as bearing on the subject under discussion.

"A gentleman, aged about forty, had been for some time losing considerable quantities of blood by arterial hæmorrhage from the rectum. Considering it as merely hæmorrhoidal, he had paid little attention to it, until his friends became alarmed by his altered appearance. From being strong and rather plethoric, he had become weak, exhausted, pale, and haggard. He had anasarca of his legs—his pulse was frequent and feeble, and much excited by the least exertion. Along with these symptoms, he was liable to strong and irregular action of the heart, and complained of giddiness, tinnitus aurium, violent throbbing in the head, and frequently of throbbing headach. On examining his rectum, a fungous tumor was found within the sphincter, on the apex of which a small artery was bleeding *per saltum*. This was tied, and there was no return of the hæmorrhage; and under the use of nourishing diet, and a liberal allowance of wine, all his other complaints disappeared. He made up so rapidly in flesh and blood, that not long after, apprehensions were entertained that he was becoming too plethoric, and it became necessary to reduce his regimen, but under these circumstances he had no return of the symptoms in his head.

"A lady, aged twenty-five, had been frequently bled on account of symptoms in the head which had supervened upon an injury. Considerable relief had followed each bleeding; but the symptoms had soon returned so as to lead to a repetition of the bleeding at short intervals, and this had been going on for several months. When I saw her, she was stretched upon a couch, her face of the most death-like paleness, or rather of the paleness of a stucco figure, her pulse very rapid and as small as a thread, her general weakness extreme. The mass of blood appeared to be reduced to the lowest point that was compatible with life, but she still complained of frequent headach, violent throbbing in the head, confusion and giddiness. It was evident that evacuations could be carried no farther, and, in consultation with a very intelligent medical man who had the charge of her, it was agreed as a last experiment to make trial of the opposite system, nourishing diet and tonics. In a fortnight she was restored to very tolerable health."
